# Medical Observations

**Published:** 1873-07

**Authors:** Fred. Horner

**Affiliations:** Virginia


### MEDICAL OBSERVATIONS.

BY FRED. HORNER, JR., M.D., OF VIRGINIA.

The State of Virginia is three hundred and seventy miles long and two hundred miles broad, and contains sixty-four thousand square miles. For convenience, it may be divided into Eastern and Western, or Piedmont and Tide-water districts, the latter including all of lower Virginia, first settled by European colonists who, subsequently, with emigrants from Pennsylvania, migrated to the country at the base and west of the Blue-Ridge Mountains, embracing a vast territory reclaimed from the Indians. In 1776, Virginia had a population in excess over that of New York of four hundred thousand. At present, the latter State exceeds Virginia by several millions.

The medical topography of the Piedmont and Tide-water districts of Virginia is marked by entirely different diseases. For example, in the former, during the winter months, typhoid fever, rheumatism and diphtheria are common; while in the latter section, during summer, cholera infantum, diarrhœa and paludal or marsh fevers prevail. The climate of Virginia for several years has been marked by unusual extremes of heat and cold. In winter and spring, northeasterly winds have proved to be a prolific cause of disease, particularly neuralgia and rheumatism. The thermometer has been as low as 7° below zero. Seas of polar air, accompanied by snow storms and by high electric tension of the atmosphere or excess of ozone, have altogether been exciting causes of sickness.

Previous to entering upon the more minute details and specification of prevalent diseases common in Virginia, it may be stated that the State Medical Society, consisting of several hundred members, has been reorganized since the war, and, as the Transactions will prove, is now in a sound working condition. Valuable contributions to medical and surgical science have been furnished to the profession through this medium. They contain the address of the President, Dr. Fauntleroy, on "Vis Medicatrix Naturæ," Dr. Edwards' address on "Medical Reform," and papers on the "Epidemics of the Piedmont and Tide-water Districts of Virginia;" a report on the "Anatomical and Physiological Differences Between the White and Black Races." Dr. F. D. Cunningham is the author of an excellent article on "Defective Vision"—a production, by the way, which will compare favorably with those of such men as Liebriech and Derby. Dr. Tibault's paper on "Paludal Fever," and Dr. Claiborne's on "Dysmenorrhœa and Diphtheria," are worthy of special commendation. This Society sends delegates annually to the American Medical Association, and has been greatly useful in completing the organization of the State Board of Health of Virginia. The late report of this Board shows that among other duties required of it by the Legislature is "to examine into and report what, in their best judgment, is the effect of the use of intoxicating liquor, as a beverage, upon the industry, happiness, health and lives of the citizens of the State, and also what legislation, if any, is necessary in the premises.

The medical colleges are located at Richmond and the University of Virginia. The latter has Professor J. L. Cabell at its head. He is the founder there of the chair of Comparative Anatomy, which he fills with great distinction and acceptance to his pupils, many now scattered all over the country.

There are various hospitals for the insane, and one has lately been incorporated by act of the Legislature for inebriates. From the reports of the former, it is shown that insanity affects more farmers and unmarried persons, male and female, than any other class of society, and that intemperance and the abuse of tobacco are causes of insanity, and that the first named, whether considered as a disease or vice, is more destructive of human life than any one single cause. It may be sincerely deprecated that the greed of the State Legislature and the courts to secure a revenue by the sale of ardent spirits and licences costs the people nineteen millions of dollars annually, and has ruined thousands who have become victims to intemperance.

The *Virginia Clinical Record* is the only medical journal in this State. A quarterly or even weekly periodical is greatly needed.

I proceed to the consideration of diseases in Virginia which have been most prevalent since the late war. These have been marked by two opposite conditions, viz.: debility and excitement. Again, endemics occur, and certain diseases, malarial and continued fevers, are more severe in cyclical periods than usual. For example, intermittent and remittent fevers affected many persons throughout the State in 1827, particularly along the rivers and in Eastern Virginia. Many died from the violence of these diseases, and perhaps from the malpractice of that period, which was to dose and bleed—a practice not authorized by the profession of the present day. These fevers were common as an epidemic in 1827–'31, and have returned since the war in 1865–'71. The failure to check the intermittent form is due frequently to the fact that quinine, which is insoluble excepting in a mineral acid, is undissolved by the gastric fluid, and acts as an irritant to the stomach. Change of residence, after the usual remedies have failed, to the mountains or to the city, will expedite the cure. The use of the thermometer to determine the relative danger and grade of symptoms in typhoid fever will assist the physician. When the degrees of 105° or 107° are attained, fatal results will ensue unless stimulants are promptly resorted to.

*Cerebro-spinal meningitis,* or *spotted fever,* a new disease whose pathology is yet to be decided by another Louis or Gerhard, was sporadic in the vicinity of Petersburg and Richmond in 1871. Among the first cases was one to which Professor Hunter McGuire was called in consultation—the attending physician had diagnosed the case to be hydrophobia. Dr. McGuire, with his wonted sagacity, detected the mistake and prescribed accordingly, and the patient was soon relieved. Spotted fever prevailed among the poorer operatives in the tobacco factories, and was fatal to many destitute of suitable shelter and food and appropriate medical treatment.

*Diphtheria*, which is a species of sore-throat exhibiting a peculiar exudation in the throat and fauces resembling tanned hide, with more or less ulceration, has been common occasionally in certain localities during the spring and winter months. It is best treated with mild aperients, Dover's powders and mucilages, followed by mineral acids, tincture of iron and quinine argent. nitras locally, and a generous diet.

*Influenza* did not fail—as Dr. Snow, of Providence, predicted—to follow in the wake of "epizooty." As usual, the prostration, acute pain in the neck and loins, fever and loss of appetite, were pathognomonic signs of this affection. It has not been confined to Virginia, but has prevailed in other parts of the country. While no positive proof can be adduced as to their identity, and that they are infectio-contagious, examples did occur tending to establish such facts. For example, in Boston, where the teamsters of the Fire Department are required to occupy the same apartment in which their horses are stabled, several men contracted epizooty, and died with all the symptoms of the disease. In this vicinity several farmers who took the precaution to isolate their horses from others that were sick had theirs to escape the disease.

*Dysmenorrhœa* is a very common affection among young females who neglect exercise in the open air, and are confined too much in heated chambers and school-rooms. Dr. Claiborne's monogram, which has been published in the first volume of the Transactions of the Virginia Medical Society, will be found to be an exhaustive treatise on this subject. Cauterization of the os uteri, preceded by dilatation and proper hygienic and medical treatment, can not be too strongly urged.

*Puerperal Peritonitis.*—This dreaded and fatal disease may be often traced to the neglect of preparatory treatment previous to child-bearing, and the too common resort, in the Southern States, to negro midwives—resulting often in the loss of the life of mother and child. I have repeatedly saved life and insured prompt delivery in cases of difficult labor, and where the nervous tension was marked and the pulse indicated depletion, by bleeding copiously from the arm and the use of ether, and thereby averted the occurrence of puerperal fever. Ligation of the funis, placental and umbilical, has proved, in my experience, the safest, not neglecting, after delivery, the application of the binder to the abdomen. A single disease to which children are liable may be cited, viz.: tuberculosis of the spine, or so-called hip or spinal disease. Our distinguished countryman, Dr. Benjamin Lee, of Philadelphia, makes this disease a specialty of surgical practice. He has succeeded, with others, greatly in relieving this class of sufferers by judicious mechanical apparatus. Recently I have seen, at the St. Thomas Hospital in London, more than two hundred children afflicted with this disease under various modes of treatment, all including the mechanical, and was assured by the Surgeon, Mr. Cline, that the practice had proved to be most encouraging. Among the number of internal remedies, cod-liver oil, bromide of potass., morphia, beef wine and iron were the most used.

In conclusion, brief reference may be made to the class of preventible diseases and injuries too often neglected by physicians. Intemperance stands at the head of the first category, while pistol and gun-shot wounds inflicted by careless, vicious or criminal persons includes some of the last mentioned. The amount of $140,-095.30 is paid out of the public treasury of Virginia per annum to defray the expense of criminal prosecutions, while $19,000,000 is expended for the purchase of the various forms of ardent spirits consumed by the people. The physician can never be absolved from the discharge of the duty, in the language of the Code of Ethics of the American Medical Association, "to warn the public as to matters of public hygiene," and it should be his highest aim to prevent disease, as well as to cure it.

---

LARYNGITIS.—Dr. Freeman remarks: "In the treatment of a case of this distressing disease which occurred recently in my own practice, after the application of leeches and fomentations, together with the administration of slightly nauseating doses of antimony and lobelia, I made free application of the cantharidal collodion over the region of the larynx; and this seemed to prepare the way for the greatest relief which the patient experienced from the careful and persistent inhalation of the steam of boiling water."

